# S-allylcysteine suppresses ovarian cancer cell proliferation by DNA methylation through *DNMT1*

**DOI:** 10.1186/s13048-018-0412-1

**Published:** 2018-05-14

**Authors:** Yasi Xu, Dan Su, Lucheng Zhu, Shirong Zhang, Shenglin Ma, Kan Wu, Qiang Yuan, Nengming Lin

**Affiliations:** 1 Hangzhou Translational Medicine Research Center, Zhejiang Chinese Medical University, Hangzhou First People’s Hospital, No.261 Huansha Road, Shangcheng District, Hangzhou, 310006 China; 2 Department of Pathology, Zhejiang Cancer Hospital, No.38, Guangji Road, Hangzhou, 310022 China; 3 Department of Oncology, Hangzhou Cancer Hospital, No.34, Yanguan Road, Hangzhou, 310002 China; 4 College of Pharmaceutical Science, Zhejiang Chinese Medical University, No.548 Binwen Road, Binjiang District, Hangzhou, 310053 China

**Keywords:** Epigenetic, DNA methylation, S-allylcysteine, Ovarian cancer

## Abstract

**Background:**

The anti-tumor effects of S-allylcysteine (SAC), a water-soluble garlic derivative, on human ovarian cancer cells have been previous studied in vitro and in vivo models but the precise epigenetic molecular mechanisms are still unclear. This study aimed to investigate the epigenetic mechanism of SAC.

**Methods:**

Human epithelial ovarian cancer cell line A2780 was selected. Cell proliferation and cell cycle was analyzed. DNA methylation, DNA methyltransferase (DNMT) activity, tumor suppressor gene expressions, as well as protein expression were analyzed.

**Results:**

SAC could inhibit the proliferation of A2780 cells in dose- and time-dependent manners (the IC_50_ was 16.25 mmol/L and 5.25 mmol/L at 48 h and 72 h). Treatment of A2780 cells with SAC resulted in G1/S phase arrest. SAC treatment decreased global DNA methylation levels in A2780 cells in a dose-dependent manner. SAC decreased the levels of 5-methylcytosine, DNMT activity, messenger RNA (mRNA) and protein levels of DNMT1. Additionally, SAC treatment resulted in re-expression of the mRNA and proteins of silenced tumor suppressor gene *CDKN1A* accompany with reduced cell division control 2 expression.

**Conclusion:**

Our data indicated the potential therapeutic effects of SAC on the human ovarian carcinoma cell line A2780 **in vitro**. The epigenetic mechanism of action of SAC may have important implications for epigenetic therapy.

## Background

For many decades, ovarian cancer has been the most common gynecologic cancer and the leading cause of cancer death worldwide [1, 2]. The prognosis of most ovarian cancer patients remains poor even after surgery and paclitaxel/platinum-based chemotherapy. This is mainly caused by drug resistance, cancer metastasis, high cancer heterogeneity, and rapid tumor progression, etc.[3, 4] Therefore, continued development of novel anticancer drugs for ovarian cancer is urgently required.

Natural compounds derived from food have been investigated as new sources of anticancer drugs [5]. These agents act in a manner similar to the conventional anticancer drugs by disrupting the cell cycle or inducing apoptosis. Garlic (*Allium sativum* L.) is an edible crop with a wide range of traditional uses in treating different ailments including cancer, diabetes, and cardiovascular diseases.[6] Large-scale epidemiological studies within the past few decades have suggested a correlation between garlic consumption and a reduced incidence of cancer.[7, 8] Further investigation showed that organosulfur compounds naturally found in garlic are likely to be responsible for the decreased cancer risk.[9] *S*-allylcysteine, a water-soluble compound derived from garlic,[10] has been demonstrated its antitumor efficacy by inhibiting proliferation and inducing apoptosis in ovarian cancer cells [11].

Epigenetics focuses on those heritable changes which do not result from changes of DNA sequence. DNA methylation, which is the addition of a methyl group to a cytosine (C) residue with the aid of an enzyme known as DNA methyltransferase (DNMT), is the most characterized epigenetic mechanism.[12, 13] Gene promoter hypermethylation has been recognized as an important mechanism by which tumor suppressor genes are shut down during development of tumors. It also has been reported that approximately half of the tumor suppressor genes are inactivated by epigenetic mechanisms rather than by genetic mechanisms in sporadic cancers. DNA methylation is commonly associated with increased levels or altered functions of DNMTs [14].

Our previous data demonstrated the potential therapeutic effects of SAC on the human ovarian carcinoma cell line A2780 in vitro [15]. SAC suppresses cell proliferation while simultaneously inducing apoptosis. In this study, we aimed to investigate the epigenetic mechanism contributing to antitumor efficacy by SAC.

## Methods

### Materials

Reangents were purchased as follows: RPMI-1640 medium and fetal bovine serum (FBS) (Thermo Scientific, South Logan, UT, USA), CCK-8 kits and Hoechst 33,258 (Sigma-Aldrich, St Louis, MO, USA), Giemsa solution (Solarbio, Beijing, China), BD BioCoat™ BD Matrigel™ Invasion Chambers, Cycletest Plus DNA Reagent and Annexin-V-Fluor Staining Kits (BD Biosciences, Franklin Lakes, NY, USA), Gentian violet (Huyu Biotech Co, Ltd., Shanghai, China), Cell Lysis Buffer (Cell signaling, Danvers, MA, USA), PVDF membrane (Millipore, Billerica, MA, USA), ECL Plus substrate (Thermo Scientific Pierce, Rockford, IL, USA), internal reference antibody against GADPH and primary antibodies against CDKN1A, cell division control 2 (CDC2), and p-CDC2 (Abcam Inc., Cambridge, MA, USA), 5-methylcytosine (5-mC) (Calbiochem, EMD Biosciences, SanDiego, CA), DNMT1, DNMT3a and DNMT3b (Imgenec Corporation, San Diego, CA), Methylamp™ Global DNA Methylation Quantification Kit and EpiQuik DNMT Activity Assay Kit (Epigentek, New York, NY), Standardized real-time polymerase chain reaction (PCR) primers for *DNMT1*, *DNMT3a*, *DNMT3b*, and *CDKN1A* (SuperArray Biosciences, Fredrick, MD).

### Preparation of SAC

SAC was purchased from Shanghai Fundamental Industrial Co, Ltd. (Shanghai, China). A 500 mmol/L stock solution of SAC was freshly prepared in phosphate-buffered saline (PBS) according to the manufacturer’s instructions and was diluted accordingly as needed.

### Cell culture

The human epithelial ovarian cancer cell line A2780 was kindly provided by the Zhejiang Cancer Hospital. The cells were cultured in RPMI-1640 medium supplemented with 10% FBS and 1% penicillin/streptomycin in a 37 °C incubator supplied with 5% CO2.

### Cell count Kit-8 (CCK-8) assay

Cells were seeded at a density of 5000 cells per well in 96-well plates in 100 μL of medium and were incubated for 48 h before treatment. The cells were treated with different concentrations of SAC for 1, 2, or 3 d. The medium was then removed, and 200 μL of fresh medium containing 5% CCK-8 was added for a further 1.5 h. The color intensity was measured using a Multiskan Spectrum spectrophotometer (Thermo Scientific, Rockford, IL, USA) at 450 nm. Each experiment consisted of eight replicates, and at least three individual experiments were performed.

### Cell cycle analysis

A2780 cells (3 × 10^5^) were cultured in 6-well plates for 48 h prior to the experiments. The cells were treated with different concentrations of SAC, ranging from 0 to 10 mmol/L, for 24 h. The cells were trypsinized and fixed with 75% ice-cold ethanol for several hours and then stained with the Cycletest Plus DNA Reagent Kit according to the manufacturer’s instructions. The DNA content of 10,000 cells was analyzed by flow cytometry for each experiment (FACSCalibur, Becton Dickinson, Franklin Lakes, NJ, USA). Each experiment was analyzed in duplicate, and at least three independent experiments were performed.

### Global DNA methylation assay

The total genomic DNA was extracted from the cells, which were treated with SAC or 5-aza-dc using the Qiagen amp^R^ DNA Mini Kit (Qiagen Sciences, Maryland, MD) following the manufacturer’s instructions. The Global DNA methylation levels were determined using the Methylamp™ Global DNA Methylation Quantification Kit according to the manufacturer’s instructions. This analysis provides the levels of global DNA methylation, and is not specific to any particular gene. The methylated fraction of DNA is recognized by a 5-mC antibody. With this colorimetric kit, the amount of methylated DNA, which is proportional to the optical density intensity, is quantified through an enzyme-linked immunosorbent assay-like reaction.

### 5-mC immunostaining

Cells were treated with various concentrations of SAC (0, 5, 10, 20 and 30 mmol/L) for 72 h and then harvested. A total of 1 × 10^5^ to 2 × 10^5^ cells were cytospun using a Cytospin 4 Equipment (Thermo Electron Corporation, Waltham, MA) at 1500 r.p.m. for 15 min and then processed for 5-mC cytostaining. Briefly, cells were permeabilized with 0.2% Triton X-100 in phosphatebuffered saline (PBS), washed with PBS for 10 min. The cells were then blocked with 3% preimmune goat serum in PBS for 30 min, followed by incubation with 3% H_2_O_2_ for 20 min to quench endogenous peroxidase. After washing the cells with PBS, cells were incubated with 5-mC-specific antibody (1:500, vol/vol; Calbiochem, Gibbstown, NJ) for 2 h, followed by sequential incubation of cells with biotinylated goat anti-mouse IgG1 and horseradish peroxidase-conjugated streptavidin and finally with diaminobenzidine substrate and counterstaining with methylene blue.

### DNMT activity assay

A2780 cells were treated for 72 h with various concentrations of SAC. After desired time point, cells were harvested and nuclear extracts were prepared from various treatment groups using Epiquik Nuclear Extraction Kit (Epigentek) following the manufacturer’s instructions. DNMT activity was determined using Epiquik DNMT Activity Assay Kit (Epigentek) according to the manufacturer’s protocol.

### Quantitative mRNA analysis of DNMTs and tumor suppressor gene *CDKN1A* using RT-PCR

Total RNA was extracted from the cells in different treatment groups using Trizol Reagents Kit (Invitrogen) and complementary DNA was synthesized through the reverse transcription reaction (iScript complementary DNA Synthesis Kit; Bio-Rad Laboratories). Using SYBR Green/Fluorescein PCR Master Mix, complementary DNA was amplified using real-time PCR with a Bio-Rad MyiQ thermocycler and SYBR Green detection system (Bio-Rad Laboratories). Samples were run in duplicate to ensure amplification integrity. Manufacturer supplied standardized primer pairs were used to measure the following: *DNMT1*, *DNMT3a*, *DNMT3b*, and *CDKN1A*. The standard PCR conditions were: 95 °C for 15 min and then 40 cycles at 95 °C for 30 s, 55 °C for 30 s and 72 °C for 30 s, as recommended (SuperArray Bioscience Corporation, Frederick, MD). The mRNA expression levels of genes were normalized to the expression level of the housekeeping gene *GADPH* and relative to the average of all delta Ct-values in each sample using the cycle threshold (Ct) method.

### Cell lysates and western blotting

Analysis of protein levels was performed using western blotting. Cell lysates from different treatment groups were prepared as described previously [14, 15]. Proteins (25–40 μg protein) were electrophoresed on premade 10% Trisglycine gels (Invitrogen) and then transferred onto nitrocellulose membranes. After blocking in freshly prepared PBS containing 3% non-fat dry milk at room temperature for 30 min, the membranes were incubated with antibodies against DNMT1, DNMT3a and DNMT3b, CDKN1A, CDC2, pCDC2, and GADPH at 4 °C overnight followed by an anti-rabbit peroxidase-conjugated secondary antibody at 1:1000 dilution (Santa Cruz Biotechnology, Santa Cruz, CA). Protein bands were visualized on X-ray film using an enhanced chemiluminescence system (Amersham Life Science, Piscataway, NJ). Equal protein loading was verified using anti-b actin antibody. Experiments were repeated three times, and thus three western blots were run in each experiment, and representative blot is shown in each case.

### Statistical analysis

Statistical analyses and data visualization were performed using IBM SPSS version 22.0 (IBM SPSS, Inc., Chicago, IL, USA) and GraphPad Prism, Version 6.01 (GraphPad Software Inc., San Diego, CA, USA). A two-tailed Student’s t-test was used for analysis of continuous variables. *P* < 0.05 was considered statistically significant.

## Results

### SAC inhibits the proliferation of A2780 cells in a dose- and time-dependent manner

**Fig. 1:**
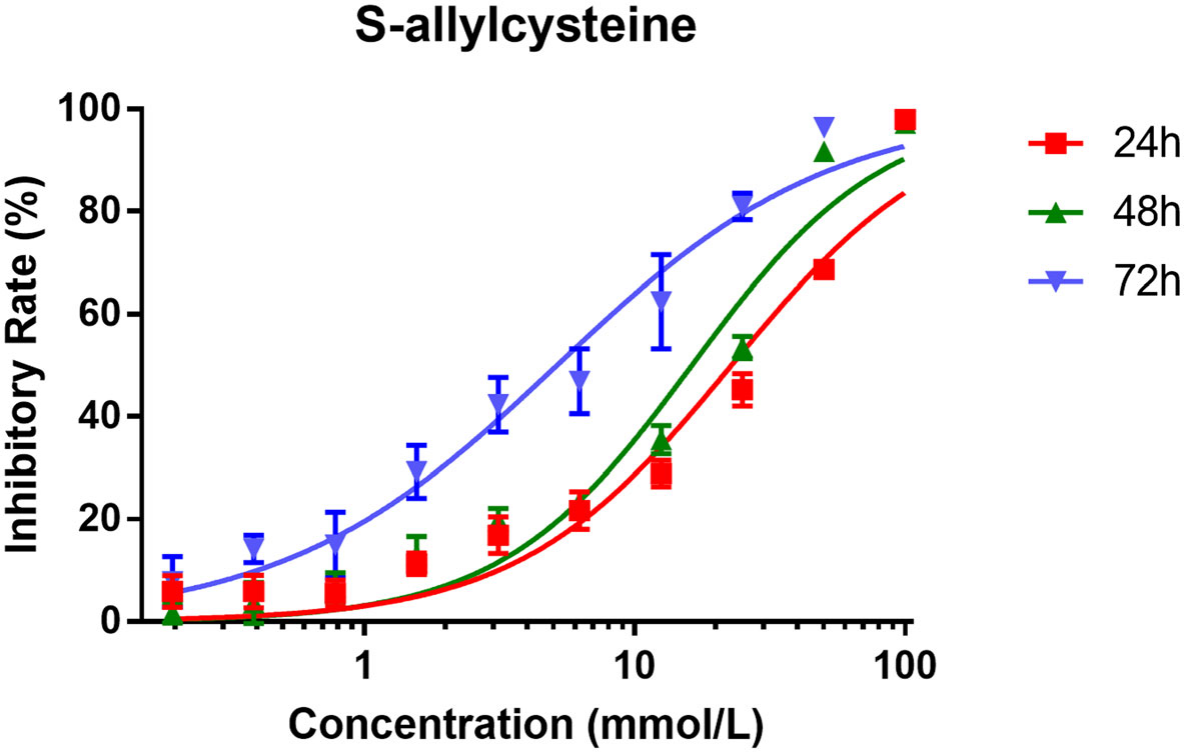
The inhibitory effect of SAC on the proliferation of A2780 cells after 24, 48, and 72 h treatment. The IC_50_ were 22.76 mmol/L, 16.25 mmol/L, and 5.15 mmol/L, respectively

A2780 cells were treated with increasing concentration of SAC and proliferation was assessed using CCK-8 assays. 12.5 mmol/L of SAC resulted in 29% growth inhibition and 50 mmol/L of SAC resulted in 69% growth inhibition after 24 h treatment (Fig. 1). The IC_50_ of SAC was 22.76 mmol/L. The results showed a dose-dependent anti-proliferative effect of SAC on A2780 cells. Next, we determined the inhibitory effects of SAC on A2780 cells at 48 h and 72 h. The IC_50_ were 16.25 mmol/L at 48 h and 5.15 mmol/L at 72 h, respectively (Fig. 1). The IC_50_ decreased corresponding to prolonged time of SAC exposure.

### SAC results in G0/G1 phase cell cycle arrest of A2780 cells

**Fig. 2:**
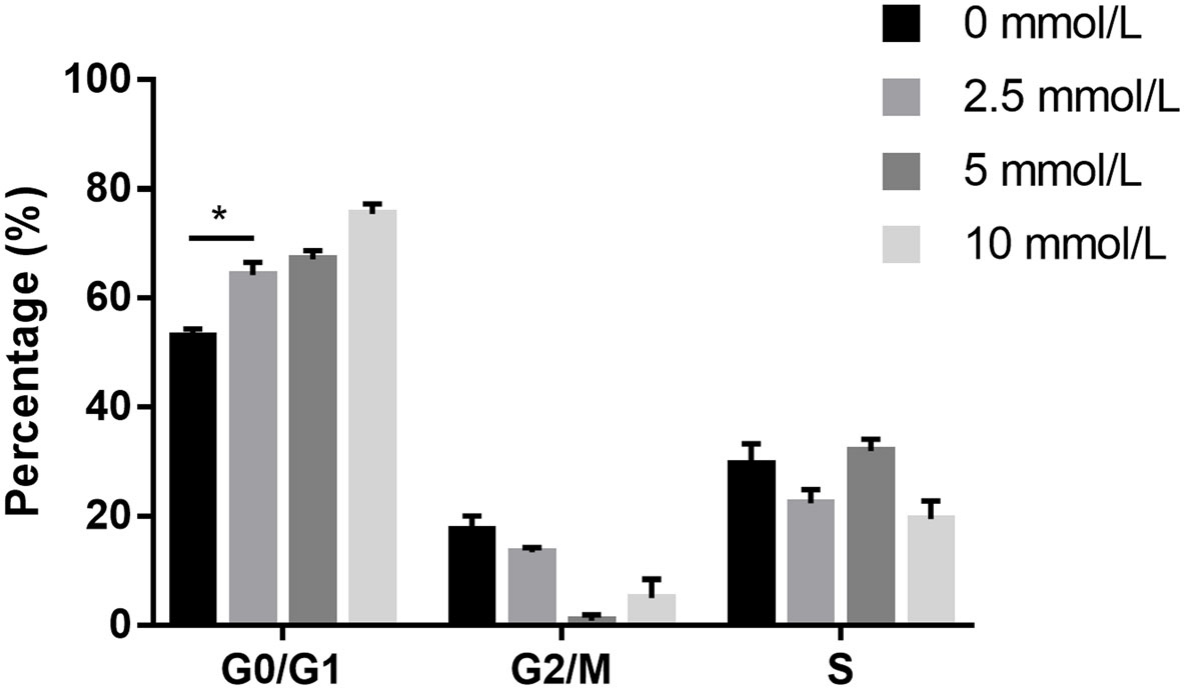
Quantitative cell cycle analysis of A2780 cells in response to SAC treatment

After treatment with SAC at a concentration of 2.5 mmol/L, flow cytometry showed a small increase in the percentage of G0/G1 phase in A2780 cells. When the SAC concentration increased to 5 and 10 mmol/L, higher percentage of G0/G1 phase was observed (*p* < 0.001). Besides, flow cytometry showed 2.5 mmol/L of SAC resulted in a small decrease in the percentage of G2/M phase in A2780 cells (Fig. 2). As the SAC concentration increased to 5 and 10 mmol/L, almost none of these cells stayed in the G2/M phase. Cell cycle changes may have caused the marked reduction in A2780 proliferation described in the previous section.

### SAC reduces global DNA methylation in A2780 cells

**Fig. 3:**
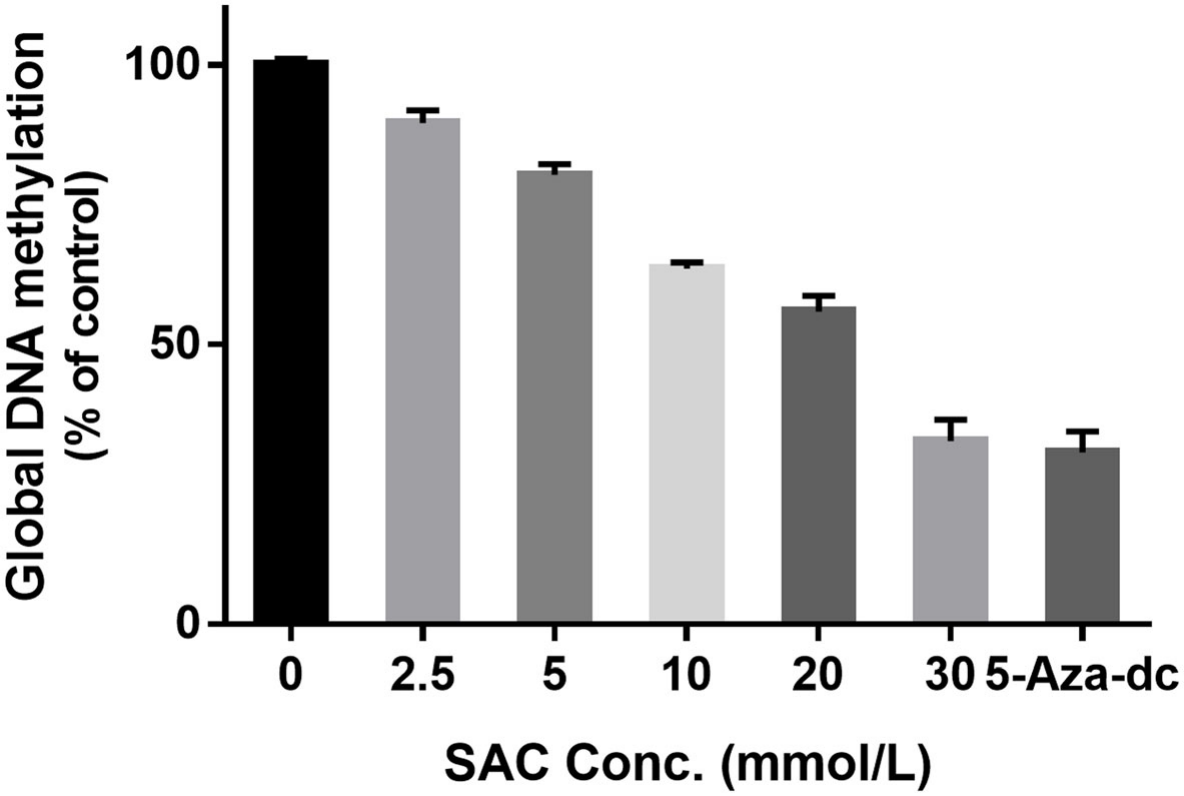
Dose-dependent effect of SAC on the Global DNA methylation levels in A2780 cells. Cells were treated with SAC for 72 h. Data are presented in terms of percent of control (non-SAC-treated) group, which was assigned a value of 100%, and as means ± SD, *n* = 3

To determine the effect of SAC on global DNA methylation in human ovarian cancer cells. A2780 cells were treated with various concentration of SAC for 72 h. Then cells were harvested and DNA was isolated for the analysis of global DNA methylation level. As shown in Fig. 3, treatment of cells with SAC resulted in a reduction in the levels of DNA methylation in a dose-dependent manner. 5-Aza-dc is a potent DNA demethylation agent and used as a positive control.

### SAC induces DNA 5-mC demethylation in A2780 cells

**Fig. 4:**
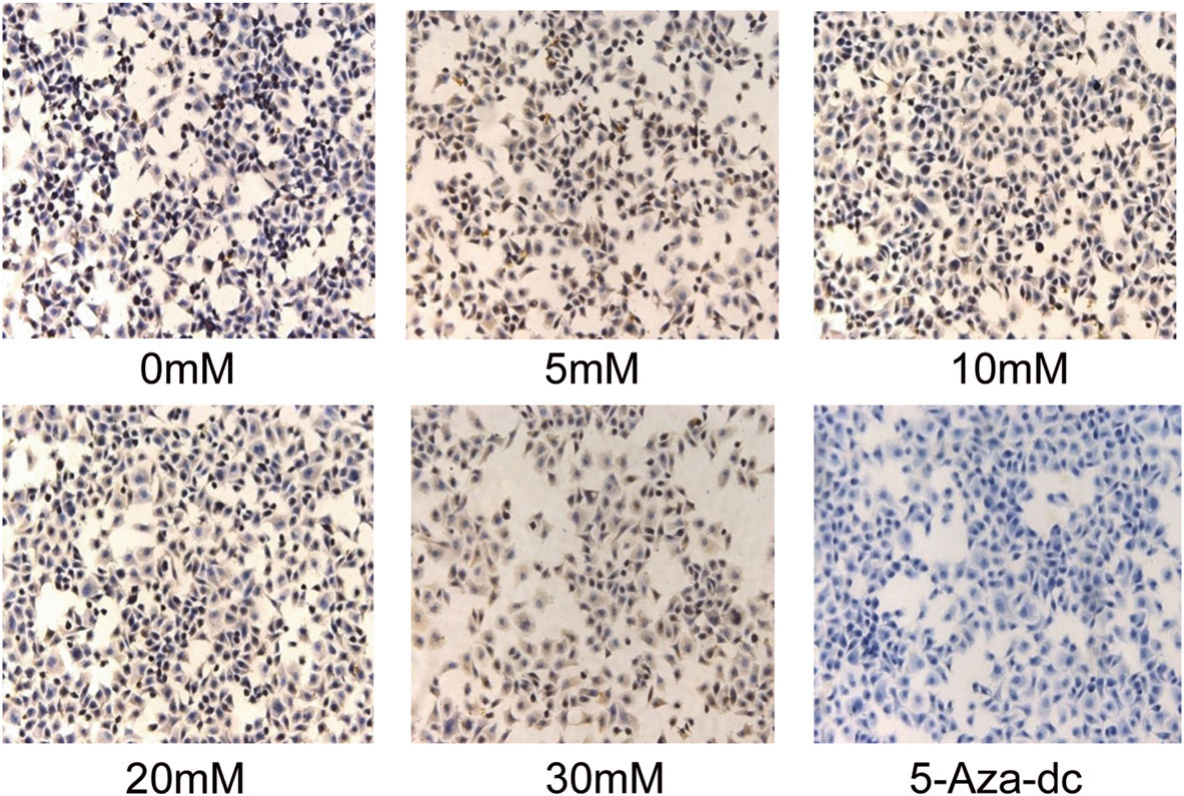
Treatment of A2780 cells with SAC for 72 h decreased the levels of 5-mC. Treatment of cells with 5-aza-dc was used as a positive control. Cytostaining of 5-mC-postive cells using a 5-mC-specific antibody

To examine the effect of SAC on DNA demethylation, A2780 cells were treated with SAC for 72 h. Cells were harvested and immunocytostaining was performed to detect 5-mc-positive cells. Cells were also treated with 5-aza-dc as a positive control. As shown in Fig. 4, SAC treatment decreased 5-mC-positive cells in a dose-dependent manner compared with non-SAC-treated A2780 cells (*P* < 0.001).

### SAC and 5-aza-dc decrease DNMT activity, mRNA and protein expression of DNMTs in A2780 cells

**Fig. 5:**
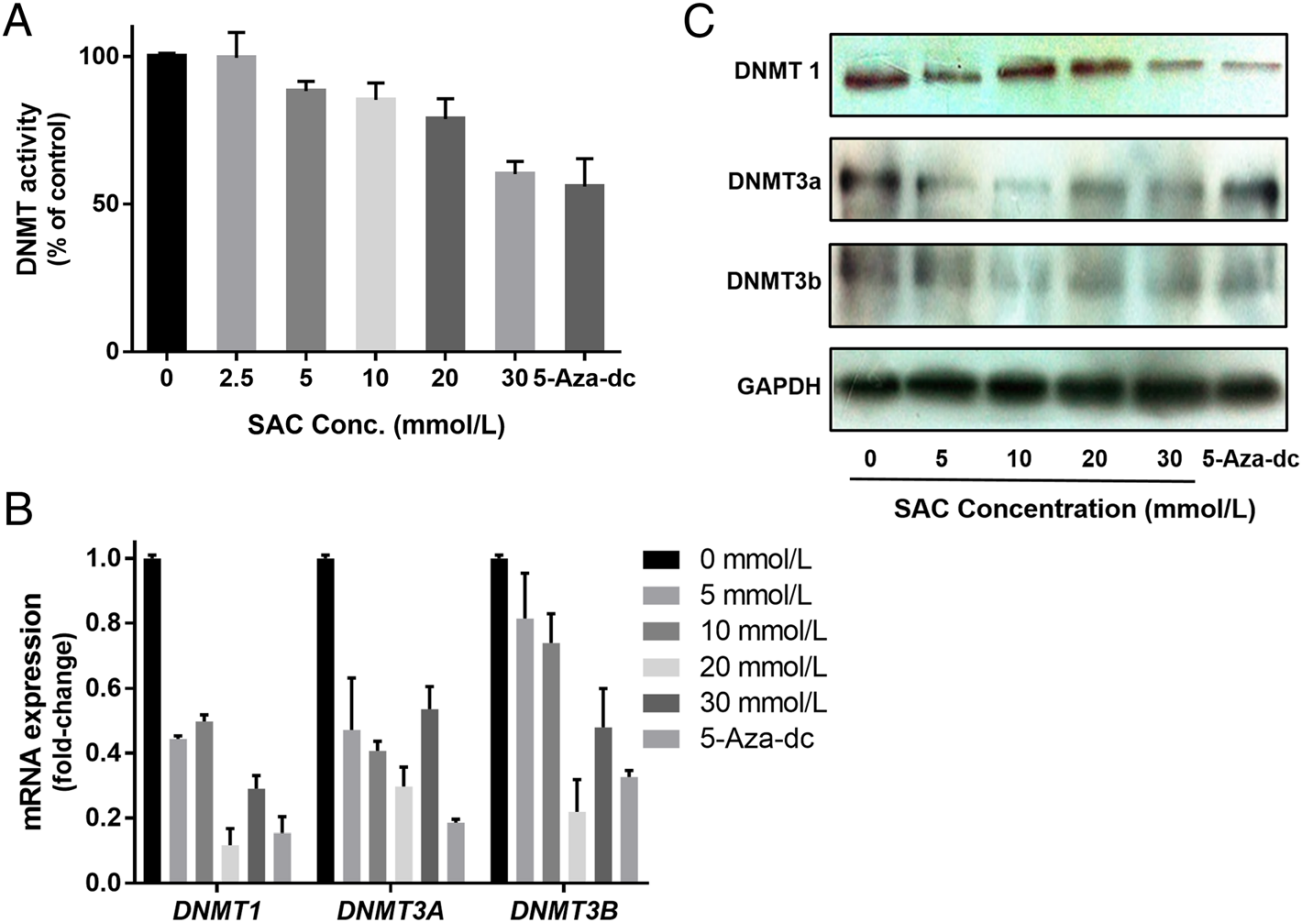
Treatment of A2780 cells with SAC or 5-aza-dc for 72 h inhibits DNMT activity and decreases the levels of mRNA and protein expressions of DNMT1, DNMT3a and DNMT3b. **a** total DNMT activity in nuclear extracts was determined using the DNMT Activity Assay Kit. Data are presented in terms of percentage versus the results using non-SAC-treated controls, which was assigned a value of 100%, and as means ± SD; *n* = 3. **b** Quantitative real-time PCR analysis of mRNA levels of *DNMT1*, *DNMT3a* and *DNMT3b* in cells. The results are presented as the expression of the individual mRNA with normalization to β-actin and as means ± SD, *n* = 3. **c** The levels of DNMT1, DNMT3a and DNMT3b in cell lysates were determined using western blot analysis after treating the cells with SAC for 72 h. The treatment groups of 5-aza-dc were used as controls

As DNMTs play a crucial role in DNA methylation, we next determined the activity of DNMT in A2780 cells after treatment of cells with SAC for 72 h. SAC decreased DNMT activity after treatment of cells in a dose-dependent manner (*P* < 0.001). The decrease in DNMT activity by SAC may be due to reduced expression of DNMT. These results were consistent with the quantitative analysis of the mRNA expression of the DNMTs using real-time PCR (Fig. 5b). The mRNA levels of *DNMT1*, *DNMT3a* and *DNMT3b* were significantly decreased (*P* < 0.001) in A2780 cells after the treatment of cells with SAC for 72 h. There was also a decrease in the protein expression levels of DNMT1, but not DNMT3a and DNMT3b after the treatment of cells with SAC for 72 h compared with non-SAC-treated controls.

### SAC and 5-aza-dc reactivate *CDKN1A* in A2780 cells

**Fig. 6:**
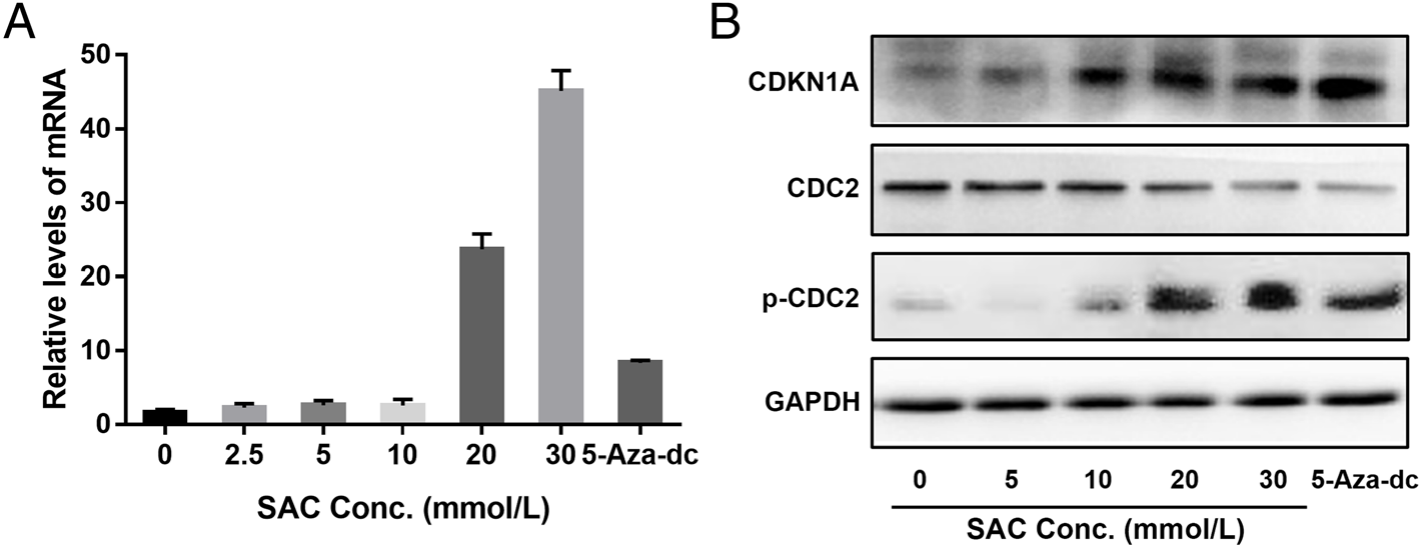
Treatment of cells with SAC or 5-aza-dc for 72 h reactivates *CDKN1A*. **a** RNA was isolated from the cells of different treatment groups and subjected to the quantification of mRNA expression levels of by *CDKN1A* RT-PCR. Data were normalized to housekeeping gene (*GAPDH*) and are presented as relative change in mRNA levels in terms of mean ± SD (*n* = 3). **b** The protein levels of CDKN1A, CDC2 and p-CDC2 were determined in cell lysates using western blotting under identical conditions

We further determined whether DNA demethylation after SAC treatment could reactivate tumor suppressor genes in A2780 cells. For this purpose, A2780 cells were treated with various concentrations of SAC and 5-aza-dc for 72 h. Cellular RNA from different treatment groups was isolated and subjected to real-time PCR as detailed under Materials and Methods. As shown in Fig. 6a, RT-PCR analysis showed that SAC treatment significantly increased mRNA levels of *CDKN1A* (*P* < 0.001) in a dose-dependent manner. 5-Aza-dc treatment also significantly increased the mRNA levels of *CDKN1A*. Similar to mRNA, protein levels were also determined using western blot (Fig. 6b). SAC treatment reactivated the silenced *CDKN1A* in a dose-dependent manner, and similar reactivation effect on tumor suppressor proteins was also observed when cells were treated with 5-aza-dc under same conditions. CDKN1A is a potent cyclin-dependent kinase inhibitor, then we detected the cyclin-dependent kinases. Western blot showed SAC downregulated CDC2 but upregulated p-CDC2.

## Discussion

This study showed SAC dose-dependently inhibited the proliferation of human ovarian cancer A2780 cells and caused cell cycle arrest in G1/S phase. Epigenetic gene regulations have been recognized to play a crucial role in the etiology of cancer. DNA methylation is an important epigenetic event in regulation of gene expression and maintenance of cellular function and that may contribute to cancer development [6, 7, 10]. Our data showed SAC decreased the levels of global DNA methylation and the expressions of DNMT1 but not DNMT3a/DNMT3b in A2780 cells. *DNMT1* is a maintenance methylase, which is the most important in the whole process of DNA methylation, whereas *DNMT3a*/*DNMT3b* is involved in de novo methylation [16]. Loo and his colleagues’ work suggest the involvement of *DNMT1* in the activation of cell cycle and DNA replication in diffuse large B-cell lymphoma cells [17]. In addition, Xiang et al. found silencing of *DNMT1* led to cell cycle arrest and promotion of apoptosis in ovarian cancer cell lines [18]. These results indicated *DNMT1* might have a correlation with cell cycle regulation. To validate this hypothesis, we determined cell cycle after different SAC exposure in A2780 cells. Similar with previous work, our data showed SAC cause G1/S arrest in A2780 cells and decreased the expression of DNMT1.

The regulation of cell cycle progression is an important system to control cancer cell proliferation. The cell cycle is a complex process that ensures correct cell division. It is tightly regulated by arrest at the G1 or G2 checkpoints and multiple molecular pathways, including oncogenic signaling, cyclin-dependent kinases (CDKs), and their regulatory inhibitors [19]. CDK inhibitor CDKN1A induces growth arrest and inhibit cell proliferation by negatively regulating cell cycle checkpoints [20, 21]. Several studies have showed *CDKN1A* could mediate G1 cell cycle arrest in cancer cells [22, 23]. Our study also found SAC reactivates or re-expresses mRNA expression of tumor suppressor gene *CDKN1A*. As the concentration of SAC increase, more fraction of G1 cell cycle arrest and CDKN1A expression were observed. As CDKN1A is a CDK inhibitor, we further detected CDK expression. Our data confirmed the expression of CDC2 was negative correlated with CDKN1A. Interestingly, increased p-CDC2 was observed when A2780 cells were treated with SAC, this might be a cellular compensation act after cell cycle arrest.

Of course, this study had some limitations: detailed mechanisms regarding how SAC regulated cell cycle, how to combination with chemotherapy or radiotherapy, etc. But we hope our data can help the validation of SAC as a potential, potent anti-cancer drug candidate.

## Conclusion

In summary, this study showed SAC could inhibit the proliferation of human ovarian cancer A2780 cells and caused cell cycle arrest in G1/S phase. SAC treatment decreased global DNA methylation levels and DNMT1 expression, and reactivated *CDKN1A*. These findings provide evidence for further understanding the anticancer mechanisms and clinical applications of SAC.
